# Risk factors for COVID-19 pneumonia in patients with hematological malignancies: a multi-center, prospective study in China

**DOI:** 10.3389/fimmu.2024.1408969

**Published:** 2024-11-07

**Authors:** Jun Li, Ran Chen, Lin Cao, Yi Liu, Yong Zhang, Xia Wei, Zhanshu Liu, Zailiang Yang, Ling Liu, Meiyu Zhou, Guofa Xu, Lanting Chen, Yao Ding, Haike Lei, Lisheng Liu, Zailin Yang, Shuang Chen, Xiaomei Zhang, Yifeng Tang, Huihui Fu, Sanxiu He, Qing Xiao, Xiaoqing Xie, Qiying Li, Yingyu Nan, Jieping Li, Xiaoliang Chen, Yao Liu

**Affiliations:** ^1^ Department of Hematology-Oncology, Chongqing University Cancer Hospital, Chongqing, China; ^2^ Mental Health Center, University-Town Hospital of Chongqing Medical University, Chongqing, China; ^3^ Department of Nuclear Medicine, Chongqing University Cancer Hospital, Chongqing, China; ^4^ Department of Hematology, the Third Affiliated Hospital of Chongqing Medical University, Chongqing, China; ^5^ Department of Hematology, Yongchuan Hospital of Chongqing Medical University, Chongqing, China; ^6^ Department of Hematology and Medical Oncology, Chongqing University Fuling Hospital, Chongqing, China; ^7^ Department of Medical Laboratory, People’s Hospital of Chongqing Liang Jiang New Area, Chongqing, China; ^8^ Department of Chongqing Cancer Multi-omics Big Data Application Engineering Research Center, Chongqing University Cancer Hospital, Chongqing, China

**Keywords:** COVID-19 pneumonia, Omicron BA.5.2, SARS-CoV-2, hematological malignancies, risk factors

## Abstract

**Purpose:**

We aimed to investigate risk factors for COVID-19 pneumonia in patients with hematological malignancies (HM) after Omicron infection.

**Methods:**

Data from a registered multi-center, prospective, observational study (ChiCTR2300071830) during the latest Omicron BA.5.2 wave in Chongqing, China was used for analysis.

**Results:**

A total of 475 HM patients enrolled in this study. COVID-19 pneumonia was observed in 15.8% (75/475) of patients, with a median age of 58 years (interquartile range [IQR], 48-69 years) and males accounting for 61.3%. Risk factors associated with COVID-19 pneumonia included: 1) Active disease status of HM at infection, with an odds ratio (OR) of 3.42 (95% confidence interval [CI]: 1.59-7.37, P=0.002) compared to complete remission (CR); 2) Incomplete COVID-19 vaccination, 1-2 doses of the vaccine (OR=2.55, 95% CI: 1.28-5.10, P=0.008) or no vaccination (OR=4.81, 95% CI: 2.45-9.43, P<0.001), as opposed to 3 doses (booster); 3) chemotherapy prior to infection, <6 months (OR=2.58, 95% CI: 1.12-5.96, P=0.027) or ≥ 6 months (OR=2.93, 95% CI: 1.31-6.53, P=0.009) compared to no chemotherapy history; 4) NK-cell reduction (< 150/μL) (OR=2.19, 95% CI: 1.27-3.79, P=0.005) versus a normal range of NK cells. During the 6-week follow-up period, 12 patients (2.5%) died, accounting for 16% of COVID-19 pneumonia patients.

**Conclusions:**

Our study investigated risk factors for COVID-19 pneumonia in HM patients after Omicron BA.5.2 infection. Highlights that HM patients with these risk factors may be susceptible to lung involvement after Omicron BA.5.2 infection and need to be taken seriously in clinical practice.

**Clinical Trial Registration:**

https://www.chictr.org.cn/bin/project/edit?pid=195998, identifier ChiCTR2300071830.

## Introduction

Since December 2021, the Omicron variant has been identified as a new variant of concern (VOC) for the severe acute respiratory syndrome coronavirus 2 (SARS-CoV-2), precipitating a renewed wave of the global pandemic (1). While the hospitalization and mortality rates associated with Omicron have seen significant reductions in the general population (2, 3), it still poses a substantial risk to patients suffering from hematological malignancies (HM) (4). According to the EPICOVIDEHA study, the mortality rate of hospitalized HM patients infected with Omicron stood at 16.5% (5), a figure markedly higher than that observed in the broader population (6–8).

The predominant cause of Omicron-related fatalities among HM patients was respiratory failure stemming from COVID-19 pneumonia. Data from the latest Omicron wave in China showed that 10.4% of HM patients develop COVID-19 pneumonia after infection (4), which was significantly higher than that in the general population (9). The reasons for this may be due to the compromised antiviral immunity resulting from the underlying disease itself, chemotherapy, or hematopoietic stem cell transplantation (HSCT) in HM patients. Nonetheless, the specific risk factors correlating with COVID-19 pneumonia development in HM patients following an Omicron infection remain insufficiently explored.

To address this gap, we analyzed data derived from a clinical study, ChiCTR2300071830. This research, spearheaded by our group, represents a multi-center, prospective observational study principally aimed at assessing antibody responses in HM patients post-Omicron infection during the latest Omicron BA.5.2 wave (spanning November 2022 to January 2023) in Chongqing, China (10). Comprehensive study details are accessible at the International Clinical Trials Registry Platform (ICTRP) associated with the World Health Organization (WHO) [https://trialsearch.who.int/].

## Methods

### Patient enrollment and follow-up

Patient enrolled in ChiCTR2300071830 Study come from 4 university hospitals in Chongqing, China. Coinciding with the emergence of the Omicron BA.5.2 pandemic in October 2022, all HM patients listed in the electronic medical records of the four participating hospitals were contacted. They were provided with an electronic informed consent form and encouraged to participate in the study should they contract a SARS-CoV-2 infection. The SARS-CoV-2 infection was confirmed by either a positive PCR result or two consecutive positive antigen tests. Detailed inclusion and exclusion criteria were described elsewhere (10). Participating patients were then tracked on a weekly basis for six weeks following their enrollment.

### Diagnosis of COVID-19 pneumonia

During the observance period, chest CT scans were carried out to diagnose COVID-19 pneumonia in patients presenting any of the following characteristics: 1) Sustained high fever (over 39.0°C) > 3 days; 2) Respiratory rate ≥ 30 beats/minute; 3) Oxygen saturation at rest ≤ 93%; 4) PaO2/FiO2 ≤ 300mmHg. The diagnosis of COVID-19 pneumonia was made independently by two experienced radiologist, and any questionable results were forwarded to a senior radiologist for expert review.

### Variables

Patient’s demographic data, diagnosis and treatment records of HM, COVID-19 vaccination history were collected as variables. Additionally, at the time of enrollment, immune function metrics were determined through counts of immune cells: neutrophils, total lymphocytes, CD4 (+) T cells, B cells, and NK cells.

### Definition of disease status among HM patients

1) Lymphoma (NHL or HL): Complete remission (CR): Defined as the disappearance of all evidence of disease, absence of symptoms, and normal imaging studies. Partial remission (PR): Defined as at least a 50% decrease in measurable disease and no new sites of disease. 2) Multiple Myeloma (MM): CR: Defined as negative immunofixation on serum and urine, disappearance of any soft tissue plasmacytomas, and less than 5% plasma cells in the bone marrow. PR: Defined as a greater than 50% reduction in serum M-protein and a reduction in 24-hour urinary M-protein by 90% or to less than 200 mg per day. 3) Acute Myeloid Leukemia (AML): CR: Defined as no evidence of disease and normalization of blood counts, no blasts in peripheral blood, less than 5% bone marrow blasts, absence of Auer rods, and no extramedullary disease. PR: Defined as meeting all hematologic criteria of CR except for bone marrow blasts >5% to <25% and a decrease of bone marrow blasts by ≥50%. 4) Chronic Myeloid Leukemia (CML): CR: Defined as a complete hematological response with no detectable Philadelphia chromosome or BCR-ABL1 transcripts. PR: Defined as a major molecular response with BCR-ABL1 transcripts >0.1% and ≤1% on the international scale. 5) Chronic Lymphocytic Leukemia (CLL): CR: Defined as the absence of lymphadenopathy, hepatomegaly or splenomegaly, and disease symptoms, normal complete blood count, and less than 30% lymphocytes in the bone marrow. PR: Defined as at least a 50% decrease in lymphadenopathy, hepatomegaly, and splenomegaly, and improvement in blood counts but not meeting the criteria for CR. 6) Myelodysplastic Syndromes (MDS): CR: Defined as bone marrow blasts <5%, absence of dysplasia, normalization of peripheral blood counts, and independence from transfusions. PR: Defined as more than a 50% reduction in bone marrow blasts compared to baseline and clinical improvement in cytopenias. Patients who have not achieved CR or PR defined as active disease (AD).

The patients were independently evaluated by two physicians according to the established criteria. In cases of disagreement, a third, senior expert was consulted to make the final decision.

### Detection of immune cell counts

Absolute counts of neutrophils and total lymphocytes were determined from peripheral blood samples using the automatic hematology analyzer XN-9000 (Sysmex, Japan). In contrast, B cells, CD4 (+) T cells, and NK cells’ absolute counts were ascertained using flow cytometry via the Beckman DXFLEX (Beckman Biosciences, USA).

### Identification of SARS-CoV-2 strains in the patients

During the pandemic, samples sourced from various city regions were sequenced by the local Centers for Disease Control (CDC). These sequences were subsequently uploaded to the Global Initiative on Sharing Avian Influenza Data (GISAID) database (https://db.cngb.org/gisaid). For our study, we selectively extracted sequencing outcomes from November 1, 2022, to January 1, 2023, to pinpoint potential SARS-CoV-2 strains in our study cohort.

### Statistical analysis

Patient characteristics were elucidated via descriptive statistics. Continuous variables were described using median and interquartile range (IQR). In contrast, categorical variables were presented as both absolute and percentage frequencies. For comparisons between the non-pneumonia and pneumonia groups, Mann–Whitney U test, Chi-square test, Chi-square test with Yates correction, or Fisher’s exact test was employed as deemed fitting. For both univariate and multivariate analyses, logistic regression models were in operation. Variables that yielded a p value ≤ 0.1 in the univariate model were included in the multivariate analysis. The optimal cutoff value for NK cell counts associated with COVID-19 pneumonia was determined using the receiver operating characteristic (ROC) curve analysis. All p values are two-sided, with a p value < 0.05 denoting statistical significance. All statistical analyses were conducted with R, version 4.2.3 (The R Foundation for Statistical Computing) and graphical representations were generated using GraphPad Prism, version 8.0.2.

## Results

### Identification of SARS-CoV-2 strains

**Figure 1:**
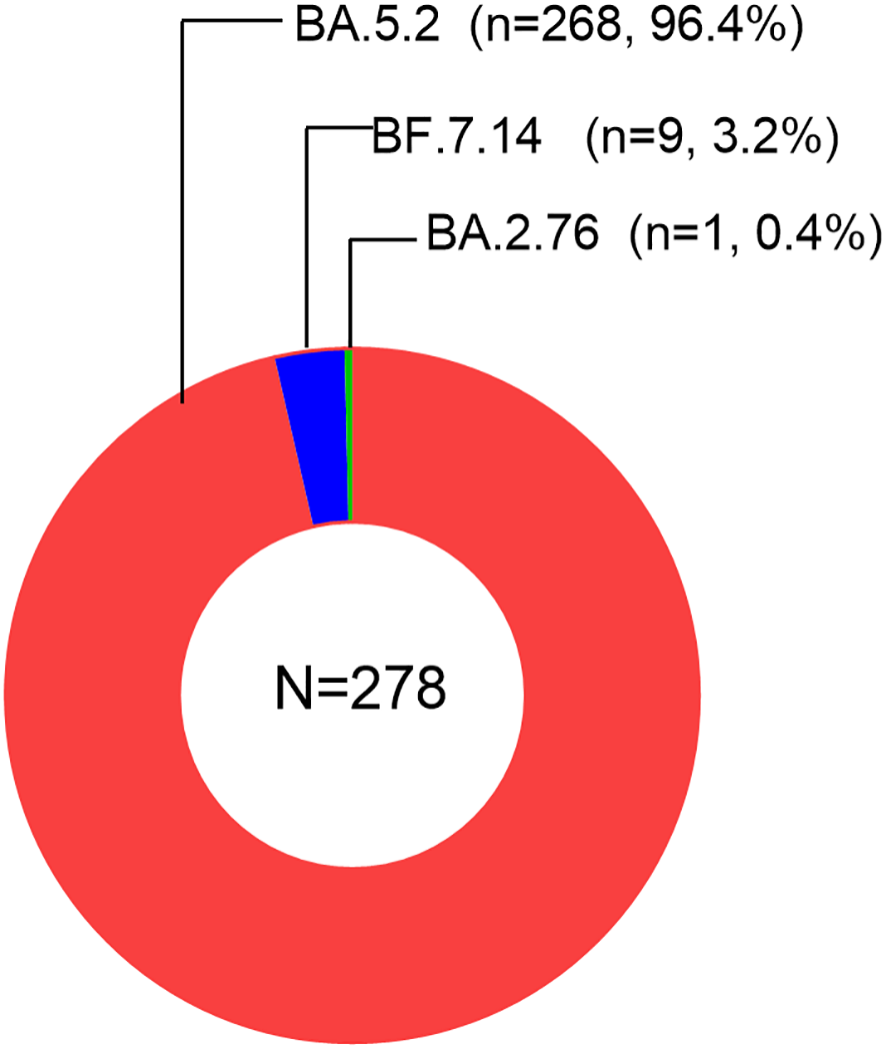
Sequencing data of 278 samples from GISAID database.

We downloaded a total of 278 sequencing data samples from the GISAID database. All the samples indicated the presence of Omicron variants. We observed the subvariant BA.5.2 of Omicron to be the most prevalent, accounting for 96.4% of the cases. This was followed by the subvariants BF.7.14 (3.2%), BA.2.76 (0.4%), as visualized in **Figure 1**. Therefore, in our study, the majority of infections were attributed to the Omicron subvariant BA.5.2.

### Patient characteristics

**Figure 2:**
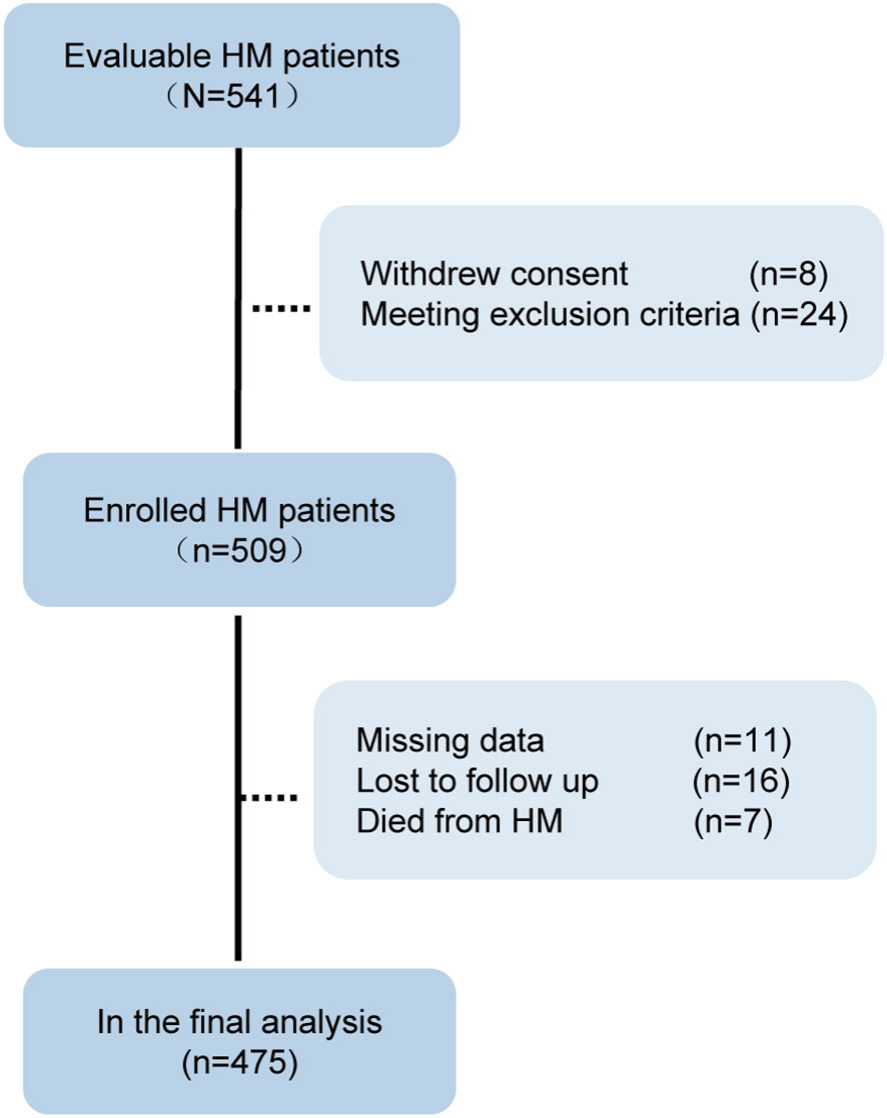
CONSORT diagram. HM, hematological malignancies.

We contacted and evaluated 541 cases, enrolling 509. After excluding 34 cases for various reasons, 475 cases were analyzed in the final dataset (**Figure 2**). The characteristics of the 475 cases were summarized in **Table 1**. Among the patients, the median age was 58 years [IQR 49-68], with a male predominance accounting for 58.7% of the total. The most common types of hematological malignancies were lymphomas (n=354, 74.5%) and multiple myeloma (n=72, 15.2%). At the time of Omicron infection, a significant proportion of patients—361 of them (76%)—had a history of COVID-19 vaccination. Additionally, 365 patients (76.8%) were reported to be either in partial remission or had active disease. Before the onset of the Omicron infection, 353 patients (74.3%) had previously undergone chemotherapy, 220 patients (46.3%) had been treated with anti-CD20 monoclonal antibody (moAb), and 41 patients (8.6%) had received autologous hematopoietic stem cell transplantation (Auto-HSCT). Immune cell count tests revealed reduced B cell counts and CD4 (+) T cell counts as the most frequently observed abnormalities in HM patients, accounting for 59.2% and 79.6% of cases respectively.

**Table 1:** Characteristics of pneumonia and non-pneumonia group.

Variables	Total	Non-pneumonia	Pneumonia	P value
	(N=475)	(N=400)	(N=75)	
Age (years), n (%)				0.996
< 40	58 (12.2)	49 (12.3)	9 (12.0)	
40-60	229 (48.2)	193 (48.3)	36 (48.0)	
> 60	188 (39.6)	158 (39.5)	30 (40.0)	
Male sex, n (%)	279 (58.7)	233 (58.3)	46 (61.3)	0.619
Baseline disease, n (%)				0.950
HL	43 (9.1)	34 (8.5)	9 (12.0)	
Aggressive B-cell NHL	183 (38.5)	157 (39.3)	26 (34.7)	
Indolent B-cell NHL	79 (16.6)	67 (16.8)	12 (16.0)	
HIV-related lymphoma	19 (4.0)	16 (4.0)	3 (4.0)	
T or NK/T-cell lymphoma	30 (6.3)	24 (6.0)	6 (8.0)	
Multiple myeloma	72 (15.2)	62 (15.5)	10 (13.3)	
Myeloid cancer ^*^	42 (8.8)	34 (8.5)	8 (10.7)	
CLL	7 (1.5)	6 (1.5)	1 (1.3)	
Disease status at infection, n (%)				0.130
Complete remission	110 (23.2)	99 (24.8)	11 (14.7)	
Partial remission	146 (30.7)	123 (30.8)	23 (30.7)	
Active disease	219 (46.1)	178 (44.5)	41 (54.7)	
Prior dose of vaccination ^#^, n (%)				<0.001
3	234 (49.3)	214 (53.5)	20 (26.7)	
1-2	127 (26.7)	105 (26.3)	22 (29.3)	
0	114 (24.0)	81 (20.3)	33 (44.0)	
Chemotherapy prior to infection, n (%)				0.014
Untreated	122 (25.7)	112 (28.0)	10 (13.3)	
<6 month	150 (31.6)	127 (31.8)	23 (30.7)	
≥ 6 month	203 (42.7)	161 (40.3)	42 (56.0)	
Anti-CD-20 moAb, n (%)	220 (46.3)	190 (47.5)	30 (40.0)	0.233
BTK inhibitor therapy, n (%)	46 (9.7)	41 (10.3)	5 (6.7)	0.340
Lenalidomide maintenance, n (%)	23 (4.8)	22 (5.5)	1 (1.3)	0.156
PD-1/PD-L1 inhibitor, n (%)	21 (4.4)	18 (4.5)	3 (4.0)	0.847
Proteasome inhibitor, n (%)	59 (12.4)	52 (13.0)	7 (9.3)	0.379
Auto-HSCT, n (%)	41 (8.6)	35 (8.8)	6 (8.0)	0.832
Neutropenia ^a^, n (%)	98 (20.6)	79 (19.8)	19 (25.3)	0.274
Lymphopenia ^b^, n (%)	232 (48.8)	193 (48.3)	39 (52.0)	0.551
B-cell reduction ^c^, n (%)	281 (59.2)	232 (58.0)	49 (65.3)	0.237
CD4 (+) T-cell reduction ^d^, n (%)	378 (79.6)	312 (78.0)	66 (88.0)	0.053
NK-cell reduction ^e^, n (%)	219 (46.1)	172 (43.0)	47 (62.7)	0.002

OR, odd ratio; CI: confidence interval; HIV, human immunodeficiency virus; NHL, non-Hodgkin's lymphoma; HIV, human immunodeficiency virus; HL, Hodgkin's lymphoma; CLL, chronic lymphocytic leukemia; moAb, monoclonal antibody; BTK, Bruton's tyrosine kinase; PD-1/PD-L1, programmed death-1/ programmed death-ligand 1; Auto-HSCT, autologous hematopoietic stem cell transplantation; COVID-19, coronavirus infectious disease 2019.

^*^Including 30 cases of acute myeloid leukemia (AML), 8 cases of chronic myelogenous leukemia (CML), and 4 cases of myelodysplastic syndromes (MDS).

^#^All patients received inactivated vaccines, including 317 (87.8%) who received Sinovac COVID-19 vaccine (CoronaVac) and 44 (12.2%) who received Sinopharm BBIBP-CorV.

a Absolute neutrophil count < 1.8×10^9^/L.

b Absolute lymphocyte count < 1.1×10^9^/L.

c Absolute B-cell count < 90/μL.

d Absolute CD4(+) T-cell count < 680/μL.

e Absolute NK-cell count < 150/μL.

### Risk factors for COVID-19 pneumonia in HM patients

Within the 475 patients, COVID-19 pneumonia was diagnosed in 15.8% of cases (N=75) at a median 8 days (range: 3-18 days) post infection. Within this subgroup, the patients had the median age of 58 years [IQR 48-69], and males comprised 61.3% of these patients. Of these, 56 patients (74.7%) had lymphomas as their primary disease. At the time of observation, 41 patients (54.7%) were in the active disease stage. In addition, 33 patients (44%) did not have a history of COVID-19 vaccination, whereas 65 patients (86.7%) had been subjected to targeted chemotherapy. In terms of immune cell reductions, B cells, CD4 (+) T cells, and NK cells were reduced in 49 patients (65.3%), 66 patients (88.0%), and 47 patients (62.7%), respectively. (**Table 1**)

**Figure 3:**
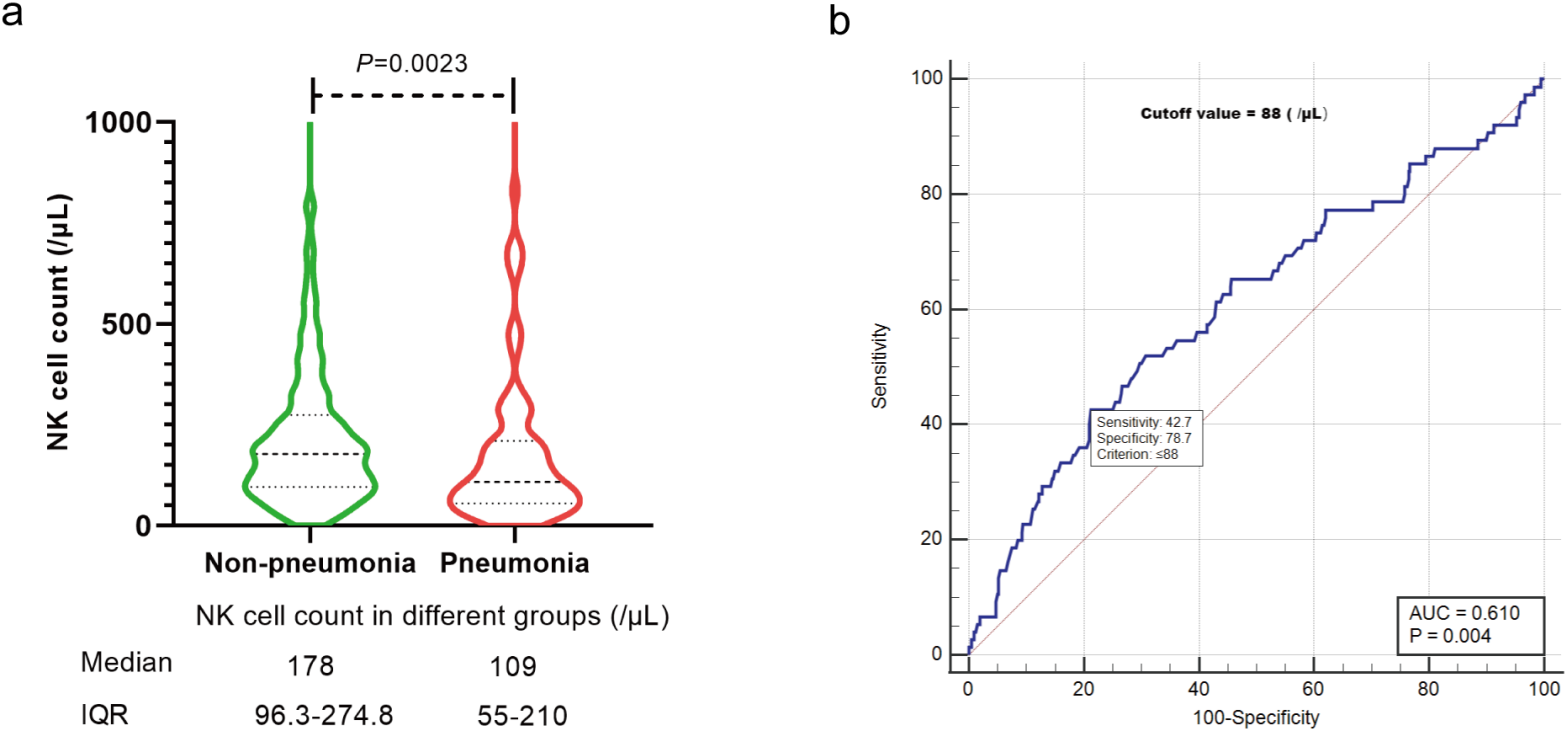
The relationship between NK cell count and COVID-19 pneumonia. **(A)** Median NK cell count in different groups. **(B)** Cutoff value of NK cell count for COVID-19 pneumonia. IQR, interquartile range.

Through multivariate analysis, we identified six variables that were significantly associated with an increased risk for COVID-19 pneumonia. The identified risk factors included: 1) Active disease status of HM at infection, with an odds ratio (OR) of 3.42 (95% confidence interval [CI]: 1.59-7.37, P=0.002) compared to complete remission (CR); 2) Incomplete COVID-19 vaccination, 1-2 doses of the vaccine (OR=2.55, 95% CI: 1.28-5.10, P=0.008) or no vaccination (OR=4.81, 95% CI: 2.45-9.43, P<0.001), as opposed to 3 doses (booster); 3) Chemotherapy prior to infection, <6 months (OR=2.58, 95% CI: 1.12-5.96, P=0.027) or ≥ 6 months (OR=2.93, 95% CI: 1.31-6.53, P=0.009) compared to no chemotherapy history; 4) NK-cell reduction (< 150/μL) (OR=2.19, 95% CI: 1.27-3.79, P=0.005) versus a normal range of NK cells. These findings are detailed in **Table 2**.We then further explored the correlation between NK cell count and COVID-19 pneumonia in HM patients. The median NK cell count was significantly higher in the non-pneumonia subgroup 178 (/μL) compared to the pneumonia subgroup 109 (/μL) (*P*=0.0023, **Figure 3A**), with a cutoff value of 88 (/μL) for predicting COVID-19 pneumonia (**Figure 3B**).

**Table 2:** Logistic regression univariate and multivariate analyses of risk factors for COVID-19 pneumonia in HM patients.

Variables	Univariable		Multivariable	
	OR (95% CI)	P value	OR (95% CI)	P value
Age, years				
< 40	1			
40-60	1.02 (0.46-2.25)	0.970		
> 60	1.03 (0.46-2.33)	0.936		
Male sex	1.14 (0.69-1.88)	0.619		
Baseline disease				
HL	1			
Aggressive B-cell NHL	0.63 (0.27-1.45)	0.276		
Indolent B-cell NHL	0.68 (0.26-1.76)	0.424		
HIV-related lymphoma	0.71 (0.17-2.98)	0.638		
T or NK/T-cell lymphoma	0.94 (0.30-3.01)	0.923		
Multiple myeloma	0.61 (0.23-1.64)	0.328		
Myeloid cancer	0.89 (0.31-2.58)	0.828		
CLL	0.63 (0.07-5.92)	0.686		
Disease status at infection				
Complete remission	1			
Partial remission	1.68 (0.78-3.62)	0.183		
Active disease	2.07 (1.02-4.21)	0.044	3.42 (1.59-7.37)	0.002
Prior dose of vaccination				
3	1			
1-2	2.24 (1.17-4.29)	0.015	2.55 (1.28-5.10)	0.008
0	4.36 (2.37-8.03)	<0.001	4.81 (2.45-9.43)	<0.001
Chemotherapy prior to infection				
Untreated	1			
<6 months	2.03 (0.93-4.45)	0.077	2.58 (1.12-5.96)	0.027
≥ 6 months	2.92 (1.41-6.07)	0.004	2.93 (1.31-6.53)	0.009
Anti-CD-20 moAb	0.74 (0.45-1.22)	0.233		
BTK inhibitor therapy	0.63 (0.24-1.64)	0.340		
Lenalidomide maintenance	0.23 (0.03-1.75)	0.156		
PD-1/PD-L1 inhibitor	0.88 (0.25-3.08)	0.847		
Proteasome inhibitor	0.69 (0.30-1.58)	0.379		
Auto-HSCT	0.91 (0.37-2.24)	0.832		
Neutropenia ^a^	1.38 (0.78-2.45)	0.274		
Lymphopenia ^b^	1.16 (0.71-1.90)	0.551		
B-cell reduction ^c^	1.36 (0.82-2.28)	0.237		
CD4(+) T-cell reduction ^d^	2.07 (0.99-4.32)	0.053	1.61 (0.72-3.59)	0.245
NK-cell reduction ^e^	2.23 (1.34-3.70)	0.002	2.19 (1.27-3.79)	0.005

OR, odd ratio; CI, confidence interval; HIV, human immunodeficiency virus; NHL, non-Hodgkin's lymphoma; HIV, human immunodeficiency virus; HL, Hodgkin's lymphoma; CLL, chronic lymphocytic leukemia; moAb, monoclonal antibody; BTK, Bruton's tyrosine kinase; PD-1/PD-L1, programmed death-1/ programmed death-ligand 1; Auto-HSCT, autologous hematopoietic stem cell transplantation.

a Absolute neutrophil count < 1.8×10^9^/L.

b Absolute lymphocyte count < 1.1×10^9^/L.

c Absolute B-cell count < 90/μL.

d Absolute CD4(+) T-cell count < 680/μL.

e Absolute NK-cell count < 150/μL.

### Outcomes of HM patients with COVID-19 pneumonia

During the 6-week follow-up period, 55 (73.3%) patients with COVID-19 pneumonia were admitted to hospital, and 24 (32%) were admitted to Intensive Care Unit (ICU) for treatment. A total of 12 patients (2.5%) died, accounting for 16% of COVID-19 pneumonia patients. Details were summarized in **Table 3**.

**Table 3:** Outcomes of HM patients with COVID-19 pneumonia during the 6 weeks follow-up.

Outcomes	N=75
Hospitalization treatment, n (%)	55 (73.3)
ICU treatment, n (%)	24 (32)
Death, n (%)	12 (16)
Related to COVID-19	7 (9.3)
Related to progressive HM	3 (4)
Related to COVID-19 and progressive HM	2 (2.7)

COVID-19, coronavirus infectious disease 2019; HM, hematological malignancies; ICU, Intensive Care Unit.

## Discussion

Since November 2021, the Omicron variant has dominated the global landscape of SARS-CoV-2 (1), with the BA.5.2 subvariant surfacing as the primary strain during the autumn/winter 2022 COVID-19 wave in Chongqing, China (10). Studies have confirmed that the severity associated with the Omicron variant is significantly lower than that of previous SARS-CoV-2 variants (12–14), with the mortality rate less than 1% in the general population (9, 11). However, in hospitalized HM patients, the mortality rate associated with Omicron is still as high as 16.5% (5), indicating that HM patients remain a significant high-risk group. Since COVID-19 pneumonia is the main cause of severe cases and death following infection (15, 16), it is crucial to study the risk factors for COVID-19 pneumonia in HM patients to ensure their protection.

Our research indicates that 15.8% (75 out of 475) of HM patients contracted COVID-19 pneumonia post-infection, with a median diagnosis time of 8 days. Concurrently, during the same Omicron outbreak in Beijing, Zhu et al. conducted a retrospective study on the incidence of COVID-19 pneumonia among HM patients infected with the BF.7 subvariant. Their findings revealed an incidence rate of 10.4% (43 out of 412) (4). Notably, our incidence rate is marginally greater than that reported by Zhu et al. Furthermore, data from the world’s most extensive retrospective analysis, the EPICOVIDEHA study, indicates that 17% of HM patients progress to critical infection following Omicron exposure (5). This figure aligns closely with our findings. However, it’s important to note that the EPICOVIDEHA study did not differentiate between specific Omicron subvariants.

To analyze the risk factors associated with COVID-19 pneumonia, we assessed patient sociodemographic characteristics, COVID-19 vaccination history, classification of HMs, disease status, history of chemotherapy, and immune function at the time of Omicron infection. Following a multivariate logistic regression analysis, the identified significant risk factors for developing COVID-19 pneumonia were: 1) Active disease status of HM at infection; 2) Incomplete COVID-19 vaccination; 3) Chemotherapy prior to infection; and 4) NK-cell reduction (< 150/μL).

To date, prospective studies investigating the risk factors for COVID-19 pneumonia in HM patients remain scarce. Our findings are juxtaposed against results from other retrospective studies for a comparative analysis. First, in our study, the term “active disease status of HM at infection” pertains to either newly diagnosed or advanced malignancies. Zhu et al. reported an overall COVID-19 pneumonia incidence of 10.4% in HM patients, however, this rate escalated to 28% and 26.7% for those with newly diagnosed HM or advanced malignancies, respectively; furthermore, their multivariate analysis denoted significant statistical differences (4). Similarly, the EPICOVIDEHA study demonstrated that an active malignancy constitutes a risk factor for Omicron-related mortality in HM patients (5, 17). These findings suggested that the disease status of HM may be related to COVID-19 pneumonia or severe illness, which was similar to ours. Second, literature suggests that HM patients require three doses of vaccines (booster) to generate adequate protective antibodies (18, 19). In alignment with this, our research posits that the absence of three doses of vaccine heightens the risk of COVID-19 pneumonia in HM patients, while EPICOVIDEHA study results concludes that three doses serve as a protective factor against severe Omicron infection. Collectively, these findings suggest that while vaccines formulated prior to the Omicron emergence appear to offer limited protection against the Omicron infection (20, 21), the trilogy of doses seems to mitigate the incidence of COVID-19 pneumonia in HM patients. This phenomenon could possibly stem from the antigenic similarities shared across different SARS-CoV-2 variants. Third, undergoing multiple chemotherapy regimens before infection, whether via traditional chemotherapy or targeted therapy, can precipitate a reduction in the quantity or functionality of immune cells in HM patients. Such treatments might also lead to a suboptimal antibody response to vaccination (22), ultimately attenuating antiviral immunity. In our dataset, 74.3% of the patients had undergone chemotherapy prior to the onset of Omicron infection. Our findings corroborate that “chemotherapy prior to infection” is a significant risk factor for developing COVID-19 pneumonia. Last, our study meticulously assessed multiple immune cell counts in the enrolled patients, encompassing neutrophils, total lymphocytes, CD4 (+) T cells, B cells, and NK cells. Upon multivariate analysis, only a reduction in NK cells (< 150/μL) emerged as a risk factor for COVID-19 pneumonia among HM patients. There may be two reasons for this. On one hand, NK-cell reduction reduces innate immune responses to viral infections. Studies found NK-cell hyper-activated (23), and exert anti-SARS-CoV-2 activity in severe COVID-19 patients (24). One the other hand, NK-cell reduction cells may also influence antibody responses to COVID-19 vaccination. For instance, NK-cell reduction can lead to attenuated secretion of interferon-gamma (IFN-γ), which in turn can curtail B cell proliferation, thereby undermining antibody synthesis (25).

However, our study has some limitations. Firstly, ethical considerations precluded us from administering chest CT scans to every participant, which might result in overlooking patients with COVID-19 pneumonia manifesting milder symptoms. Secondly, certain subgroups treated with specific targeted drugs, like the PD-1 inhibitor cohort, are underrepresented. Thus, interpreting results from these minor subsets warrants caution and calls for validation via more extensive sample sizes. Third, due to the study being conducted during the Omicron pandemic in China, the limited availability of antiviral medications meant that patients could not receive timely treatment after diagnosis. As a result, we were unable to evaluate the impact of different antiviral drugs on the occurrence of COVID-19 pneumonia following Omicron infection. Fourth, the long-term outcomes for COVID-19 pneumonia patients (such as long COVID and the duration of viable virus shedding) remain unclear because the limited follow-up period of six weeks.

## Conclusion

In our study, we identified a prevalence of 15.8% for COVID-19 pneumonia among HM patients infected with the Omicron BA.5.2 subvariant. Our analysis discerned six risk factors that appear to be significantly associated with the onset of COVID-19 pneumonia. The results underscore that HM patients exhibiting these risk factors could be predisposed to pulmonary complications post Omicron BA.5.2 infection, necessitating vigilant attention in clinical settings.

## Data Availability

The raw data supporting the conclusions of this article will be made available by the authors, without undue reservation.
